# Using Tacrolimus in Living Donor Liver Transplantation Recipients with High Model for End-stage Liver Disease Scores Might Increase the Risk of Postoperative Neuropsychologic Deficits

**DOI:** 10.12669/pjms.294.3797

**Published:** 2013

**Affiliations:** 1 Hongyu Li, Department of Liver and Vascular Surgery, Center of Liver Transplantation, West China Hospital, Sichuan University, Chengdu 610041, Sichuan Province, China.; 2 Bo Li, PhD, MD, Department of Liver and Vascular Surgery, Center of Liver Transplantation, West China Hospital, Sichuan University, Chengdu 610041, Sichuan Province, China.; 3 Yonggang Wei, MD, Department of Liver and Vascular Surgery, Center of Liver Transplantation, West China Hospital, Sichuan University, Chengdu 610041, Sichuan Province, China.

**Keywords:** Neuropsychologic deficits, Model for end-stage liver disease score, Living donor liver transplantation, Tacrolimus

For decompensated liver cirrhosis and hepatocellular carcinoma, liver transplantation provides these patients the only curative treatment option with better long-term results. The Model for End-stage Liver Disease (MELD) system is a good tool to predict short-term mortality among these patients and has also been applied to allocate liver grafts to patients on waiting lists in several countries. In 2002, the New York State Committee on Quality Improvement in Living Liver Donation prohibited live liver donation for potential recipients with MELD scores greater than 25. Alves et al suggested MELD scores >18 was associated with higher probability of graft failure after living donor liver transplantation (LDLT). Previous studies have demonstrated that using tacrolimus as primary immunosuppressant, neuropsychologic deficits had been found to be higher in recipients with elevated preoperative MELD scores, especially for those MELD scores greater than 15.^,^ We here summarize our experiences on postoperative neuropsychologic deficits in LDLT recipients using tacrolimus with high MELD scores.

From 2004 to 2012, 218 LDLT were performed in our transplant center. Postoperative neuropsychologic deficits were observed in 8 male recipients (3.7%) during the first week, including: insomnia (2), vivid dreams (1), paresthesias (1), anxiety (1), agitation (2), and cognitive impairment (1). The mean age was 42 (33-57) and the mean MELD score was 21 (9-28). All of the patients did not have the history of excessive alcohol use and preexisting central nervous system damage. Decompensated liver cirrhosis caused by HBV infection was seen in 5 patients, while hepatocellular carcinoma (HCC) was seen in 3 patients. Right living lobe liver transplantations were performed by the same medical team, with a mean graft to body weight ratio (GBWR) 0.78 (0.6-1.0). Our center used tacrolimus as the initial immunosuppressant for all liver transplant recipients. In the first postoperative week, the mean tacrolimus blood level was 9.3 (6.5-11.6), while the mean total bilirubin blood level was 23mmol/L (15-30mmol/L), the mean creatinine blood level was 67umol/L (45-90umol/L), and the mean serum sodium level was 139mmol/L (136-143mmol/L). The mean ICU stays was 9 days (7-12 days). After switching tacrolimus to sirolimus, all recipients were relieved from neuropsychlologic deficits and discharged eventually without primary graft non-function, renal failure, acute rejection, re transplantation or death.

All the 8 recipients had a relatively higher MELD scores to other LDLT recipients. Although the mechanistic pathway resulting in these neural deficits is not clear, our hypothesis is that several reasons might contribute to it: 1) elevated preoperative MELD scores usually represents a poor liver function, potential encephalopathy risk, and increased susceptibility to blood-brain barrier disruption, associating with increased vulnerability to postoperative tacrolimus neurotoxicity; 2) when GBWR <0.8, a small-for-size (SFS) living graft recipients have significantly decreased tacrolimus dosage requirements compared with non-SFS grafts recipients in LDLT. A regular dosage may cause higher tacrolimus blood levels in these SFS graft recipients and elevated blood levels of tacrolimus are associated with neurotoxicity; 3) in order to ensure the donor safety, a SFS graft may be transplanted to a high MELD recipient. This interaction may apparently increase the postoperative neuropsychologic deficits risk in such group of patients.

Although the switching from tacrolimus to sirolimus can effectively relieve patients’ neuropsychlologic deficits, there are potential risks in graft primary non-function, infection, and acute rejection. Postoperative neuropsychlologic deficits should be paid more attention when LDLT is performed for high preoperative MLED scores recipients. A lower tacrolimus dosage or a sirolimus-based immunosuppressant may decrease such risk.

## Notes

***Abbreviation:*** **MELD:** Model for End-stage Liver Disease; **LDLT:** living donor liver transplantation; **HCC:** hepatocellular carcinoma; **GBWR:** graft to body weight ratio; **SFS:** small-for-size; **PT:** prothrombintime; **ATP:** adenosine triphosphate.

## Notes

***Disclosure of Financial Interests and Potential Conflicts of Interest: ***Hongyu Li, Bo Li and Yonggang Wei have No benefits in any form nor any benefits will be received in future from a commercial party related directly or indirectly to the subject of this manuscript.
